# Billing by residents and attending physicians in family medicine: the effects of the provider, patient, and visit factors

**DOI:** 10.1186/s12909-018-1246-7

**Published:** 2018-06-13

**Authors:** Morhaf Al Achkar, Seema Kengeri-Srikantiah, Biniyam M. Yamane, Jomil Villasmil, Michael E. Busha, Kevin B. Gebke

**Affiliations:** 1 Department of Family Medicine, University of Washington, 314 NE Thornton Place, Seattle, WA 98125 USA; 2 Department of Family Medicine, Indiana University, 1110 W Michigan St #200, Indianapolis, IN 46202 USA; 3 Department of Economics, Indiana University, 100 S Woodlawn Ave, Bloomington, IN 47405 USA; 4 Western Michigan University Homer Stryker MD School of Medicine, 300 Portage Street, Kalamazoo, MI 49007 USA

**Keywords:** Billing and coding, Residency training, Revenue, Patient safety

## Abstract

**Background:**

Medical billing and coding are critical components of residency programs since they determine the revenues and vitality of residencies. It has been suggested that residents are less likely to bill higher evaluation and management (E/M) codes compared with attending physicians. The purpose of this study is to assess the variation in billing patterns between residents and attending physicians, considering provider, patient, and visit characteristics.

**Method:**

A retrospective cohort study of all established outpatient visits at a family medicine residency clinic over a 5-year period was performed. We employed the logistic regression methodology to identify residents’ and attending physicians’ variations in coding E/M service levels. We also employed Poisson regression to test the sensitivity of our result.

**Results:**

Between January 5, 2009 and September 25, 2015, 98,601 visits to 116 residents and 18 attending physicians were reviewed. After adjusting for provider, patient, and visit characteristics, residents billed higher E/M codes less often compared with attending physicians for comparable visits. In comparison with attending physicians, the odds ratios for billing higher E/M codes were 0.58 (*p* = 0.01), 0.56 (*p* = 0.01), and 0.63 (*p* = 0.01) for the third, second, and first years of postgraduate training, respectively. In addition to the main factors of patient age, medical conditions, and number of addressed problems, the gender of the provider was also implicated in the billing variations.

**Conclusion:**

Residents are less likely to bill higher E/M codes than attending physicians are for similar visits. While these variations are known to contribute to lost revenues, further studies are required to explore their effect on patient care in relation to attendings’ direct involvement in higher E/M-coded versus their indirect involvement in lower E/M-coded visits.

## Background

Given the complexity of the health care system, practice management in residency clinics can be challenging [1–5]. The salaries of physicians and, sometimes, the vitality of training programs depend on revenues generated by clinical work performed and billed for by residents [6–8]. Residency clinics in primary care specialties function under the Medicare primary care exception rules. According to these rules, an attending physician should not supervise more than four residents at once. In addition, during residents’ first 6 months of training, attending physicians must be physically present at every patient visit. After that, an attending physician needs only to provide indirect supervision to residents for preventative visits, new patient visits with evaluation and management (E/M) levels 99, 201–3, and established patient visits with E/M levels 99, 211–3. On the other hand, an attending physician must be directly involved in the care of any established patient for the visit to be coded at a higher complexity E/M levels, such as 99, 214–5 and 99, 204–5 [9]. Aside from ensuring proper compensation for clinical services, accurate coding and billing reflect the appropriate assessment of medical condition complexity and the sufficient involvement of supervising attending physicians [10].

Previous studies have highlighted inaccuracies in residents’ billing and coding [11]. Evans et al. queried 16 program directors to collect aggregate billing counts for faculty and residents in each class [12]. The cross-sectional study covering a six-month period found that residents deviated markedly from the benchmark for higher complexity E/M service code levels [12]. This pattern of residents using lower E/M codes is not limited to family medicine; Dezfuli and Smith compared resident billing to Medicare normative data and also documented higher percentages of level 3 E/M codes among resident billings.^2^ Both studies claimed significant losses of revenue [2, 12].

Practice management literature is rich with educational strategies for improving billing practices among residents. These strategies include didactic sessions [13, 14], case presentations by residents and chart audits with coders [15], workshops [11, 16], completing mock charge tickets with attendings [1], and coding checklist implementation [8]. The common assumption here is that residents’ billing and coding practices represent a competency that can be remediated through more knowledge and skill training. However, the medical education and practice literature has shown that, additionally, numerous intrinsic and extrinsic factors influence the practice of residents and doctors [17–19]. In theory and practice, provider factors (gender, position, etc.), patient factors (age, complexity, etc.), and contextual factors (time, norms, etc.) all influence learning and performance related to doctors’ tasks [17–19]. To our knowledge, no prior study has considered the potential effects of these factors on variations in resident billing practices.

Our study aims to provide a robust understanding of billing patterns in family medicine residency and explore the effects of provider, patient, and contextual factors. We analyzed six years of administrative billing data in a longitudinal study comparing individual residents’ outpatient billing codes with those used by supervising attending physicians, after controlling for visit, patient, and provider characteristics.

## Methods

### Settings

The study was conducted at a university-based family medicine residency program in the Midwest in the United States. In this program, residents and attending physicians provide full-scope family medicine services at the same outpatient clinic site, which is centrally situated in a metropolitan area. Residents’ schedules are designed to include the care for 4–6, 7–10, and 12–13 patients in their first, second, and third years of training, respectively. The clinic functions under the Medicare primary care exception rule.

### Practice management curriculum

Practice management training, which emphasizes billing education, is included in the residency program curriculum. Residents receive billing and coding education during the orientation month, as well as periodically throughout the program’s subsequent 3 years. More specifically, the curriculum includes 4 h of a “boot camp” introduction to coding and billing during the resident’s orientation, followed by another 2 h of instruction during the second year. Monthly, 1-h, small-group classes are led by faculty, as well as one-on-one chart audits with a coding specialist. In 2014, the one-on-one chart audits were replaced by a faculty-led audit of 20 charts every month.

### Data

We used the administrative record of the billing data from the family medicine residency clinic. The data consisted of the following variables: the patient ID, invoice number, day of service, patient age at day of service, procedure code and name, modifier, treatment diagnosis 1–4, and service provider. The data for this period were collapsed to define three groups of residents, namely first, second, and third year residents, and a group of attending physicians. The prevalence of visits where a 25 modifier is recorded was identical between the faculty and residents, and these visits were excluded since they indicate multiple services at the same time. The Indiana University Institutional Review Board (IRB) exempted the study protocol from further review.

### Statistical analysis

We employed logistic regression to compare billing between the faculty and residents, while delineating how patient and service provider characteristics confounded billing variation. A binary variable was constructed by grouping the higher E/M service level codes (99, 214 and 99, 215) under the value of 1, while the lower E/M service level codes (99, 212 and 99, 213) were assigned a value of 0. The likelihood of choosing higher E/M codes was denoted as an outcome variable, and explanatory variables that could potentially confound the decision making were listed. The included explanatory variables were as follows: the number of visits per year, patient’s age, provider’s gender, number of diagnoses per visits, 16 different categories of International Classification of Diseases 9th revision (ICD-9) codes, indicator variables for the resident’s year of training, and indicator variables for the resident’s class.

We used seven model specifications for our analysis. In model 1, we reported univariate regression results comparing residents (as a single group) with attending physicians, while the models 2 and 3 specified residents by year of training and class, respectively. In model 4, we included gender, age, and the number of diagnoses provided for the patient. In addition to the three characterizations in model 4, model 5 comprised fixed effects from the ICD-9 categories’ indicator variables. Models 5–7 used the same specifications as model 4, while including the residents as a single group, by year of training, and by class. Furthermore, we used stratified analysis to delineate whether the observed billing patterns were the same across the strata of analysis. We stratified by the patient’s age (≤ 18, 18–55, and ≥ 55 years), number of diagnoses listed per visit [1–4], and provider’s gender. Finally, to explain some of the variations in billing, we compared the effects of the gender, role, and rank in training. In this comparison, we used the same specifications as those in models 5–7, while dividing the sample into subgroups. Further sensitivity analysis is also reported by specifying model procedure codes as count data instead of binary variables and estimating a Poisson regression in the eighth model.

## Results

### Do residents bill differently compared with faculty?

**Fig. 1:**
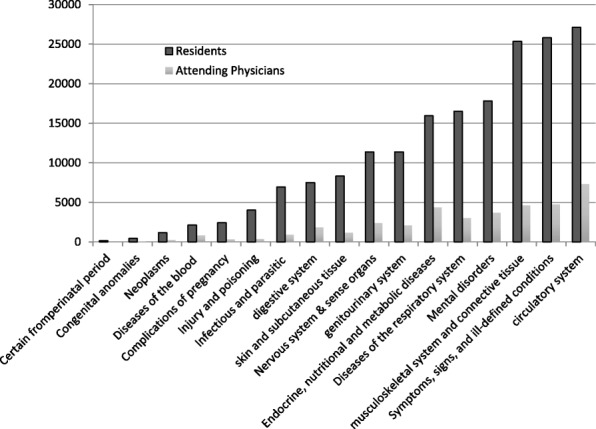
Distribution of Patient Conditions Between Residents and Attending Physicians. Note: The distribution of patient diagnoses is based on ICD classification

Table 1 shows the descriptive statistics of the data. The data spans the period between January 1^st^, 2009 and September 24th, 2015. From these data, we sampled the 98,601 visits billed as 99,212, 99,213, 99,214, and 99,215. These data represent the established outpatient visits for 116 residents and 18 attending physicians. Patients seen by residents were generally younger (40.59 vs. 47.82 years, *p* < 0.01) and had fewer reported diagnoses per visit (2.10 vs. 2.77 diagnoses, *p* < 0.01) compared with those seen by attending physicians. However, the distributions of health conditions were relatively similar between the two groups (Fig. 1).

**Table 1:** Descriptive statistics

Column1	99,212	99,213	99,214	99,215	Total
Rank					
Attending	208	11,299	12,486	312	24,305
First year resident	342	5779	3244	11	9376
Second year resident	707	15,474	6063	23	22,267
Third year resident	650	32,803	11,975	37	45,465
Gender					
Female	1079	33,051	14,654	84	48,868
Male	723	30,379	18,334	297	49,733
Number of Diagnoses					
1 diagnosis	1319	28,587	2648	13	32,567
2 diagnoses	377	21,511	5839	23	27,750
3 diagnoses	78	8188	9288	44	17,598
4 diagnoses	28	5144	15,213	301	20,686
Class year					
Class 2009	49	1548	828	2	2427
Class 2010	155	6732	2578	7	9472
Class 2011	241	6593	3493	8	10,335
Class 2012	232	7391	3452	10	11,085
Class 2013	229	7624	3080	5	10,938
Class 2014	319	7421	2034	12	9786
Class 2015	222	8816	2429	17	11,484
Class 2016	91	4272	1688	3	6054
Class 2017	56	1627	846	3	2532
Class 2018	0	107	74	2	183
Patient age group					
Age < =18	739	12,635	1814	8	15,196
Age 18–55	827	34,565	17,644	176	53,212
Age > 55	236	16,230	13,530	197	30,193

**Fig. 2:**
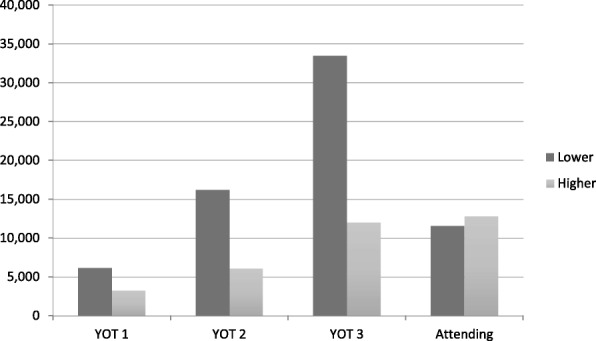
E/M Billing Patterns for Residents and Attendings. Note: YOT 1, YOT 2 and YOT 3 refer to first, second and third year residents. Lower refers to low complexity E/M codes (99212-3) while Higher refers to the high complexity codes (99214-5)

Figure 2 presents unadjusted E/M billing patterns for residents and attendings by class. In every class, residents coded fewer higher E/M level visits than the attending physicians did. Table 2 presents the results of the seven regressions models. The univariate regression (Model/Column 1) showed that, before adjusting for covariates, residents were 66% less likely to choose higher E/M codes; in other words, the attending physicians were 2.94 times more likely to bill higher E/M codes than the residents were. There appeared to be an incremental increase in deviation away from the billing pattern of attending physicians as residents advanced in training (Column 2). First-, second-, and third-year residents were 46, 61, and 75% less likely to bill higher E/M codes compared with attending physicians, respectively. This pattern of billing lower codes appears to be consistent across every class for 2009–2018 (Column 3).

**Table 2:** Regression models

Variable	Model 1	Model 2	Model 3	Model 4	Model 5	Model 6	Model 7	Model 8
Resident	0.34 (0.01)**			0.6 (0.01)**	0.55 (0.01)**			0.77 (0.01)**
Third year residents		0.35 (0.01)**				0.58 (0.01)**		
Second year residents		0.39 (0.01)**				0.56 (0.01)**		
First year residents		0.56 (0.01)**				0.63 (0.02)**		
Class 2018			0.64 (0.10)**				0.51 (0.10)**	
Class 2017			0.45 (0.02)**				0.49 (0.02)**	
Class 2016			0.35 (0.01)**				0.44 (0.02)**	
Class 2015			0.24 (0.01)**				0.36 (0.01)**	
Class 2014			0.24 (0.01)**				0.27 (0.01)**	
Class 2013			0.35 (0.01)**				0.77 (0.02)**	
Class 2012			0.41 (0.01)**				0.63 (0.02)**	
Class 2011			0.46 (0.01)**				0.88 (0.03)**	
Class 2010			0.34 (0.01)**				0.76 (0.03)**	
Class 2009			0.47 (0.02)**				2.52 (0.14)**	
Age				1.03 (0.00)**	1.03 (0.00)**	1.03 (0.00)**	1.03 (0.00)**	0.999 (0.01)**
No. of diagnosis				3 (0.03)**	2.26 (0.03)**	2.27 (0.03)**	2.43 (0.03)**	1.07 (0.01)**
Female				0.9 (0.02)**	0.95 (0.02)**	0.95 (0.02)**	0.81 (0.01)**	0.993 (0.01)**
Diagnoses Fixed Effect	NO	NO	NO	NO	YES	YES	YES	Yes
N	98,601	98,601	98,601	98,601	98,601	98,601	98,601	98,601

*p < 0.05; **p < 0.01

Columns 4–7 show the results from varying regression models, including covariates, such as patient gender, age, and the number of diagnoses listed by the provider. The adjustments in our models show that our main result—less frequent overall billing of higher E/M codes by residents than attendings, is fairly stable. Column 4 shows that, after controlling for patient age, gender, and number of diagnoses, residents are 45% less likely to bill higher E/M codes, with a slight increase to 45% when the fixed effect by diagnosis type is included in the model (Column 5). When disaggregated by year of training (Column 6), the first-, second-, and third-year residents are less likely to bill higher E/M codes by 37, 44, and 42%, respectively, compared with attending physicians; these differences are narrower than initially estimated in Column 2. Column 7 shows an overall stability of the direction of billing pattern across all cohorts except one (class 2009).

In Column 8, we report the result from a Poisson regression of the preferred E/M code. The results indicate that, residents bill higher complexity codes 0.77 times that of attending physicians (*p* < 0.01).

### Are these billing patterns limited to certain patient or provider characteristics?

We conducted stratified analysis by residents’ gender, patients’ age groups, and number of diagnoses per visit. Table 3 shows that, compared with attendings, residents were 52, 45, and 17% less likely to choose higher E/M codes when caring for older, middle-aged patients, and patients 18 years of age and younger, respectively. Residents were 45, 42, 27, and 48% less likely to choose higher E/M codes for patients with 1, 2, 3, and 4 diagnoses, respectively. When compared with the faculty as a single group (regardless of faculty gender), male and female residents were 56 and 41% less likely to choose higher bills. We also explored the interaction between gender (male vs. female) and role (attending vs. resident). A male attending physician selecting higher E/M codes is 2.27 times greater than that of a male resident. In contrast, a female attending physician is 11% *less* likely to select higher codes compared with a female resident. In addition, a male attending physician is approximately twice *more* likely to select higher codes compared with a female counterpart, while a male resident is 26% *less* likely to select higher E/M codes compared with his female counterpart.

**Table 3:** Stratified analysis

Characteristic	Odds ratio	*p*-value	N
By patients’ age			
Patient age > =55	0.48	(0.02)**	30,193
Patients age 18–55	0.55	(0.01)**	56,448
Patients <=18	0.83	(0.06)	15,196
By number of diagnoses			
One diagnosis	0.55	(0.03)**	32,567
Two diagnoses	0.38	(0.01)**	27,750
Three diagnoses	0.73	(0.03)**	17,598
Four diagnoses or more	0.52	(0.02)**	20,686
By resident’s gender			
Male	0.44	(0.01)**	56,917^a^
Females	0.59	(0.01)**	65,989^a^

*p < 0.05; **p < 0.01; ^a^includes all attendings

## Discussion

Residents’ billing patterns differ from those of attending physicians. Our study confirms this finding and assures its robustness, even after accounting for patient complexity, represented here by age, medical conditions, and number of diagnoses. Furthermore, this finding proved to exist across years of training and in 9 out of 10 classes. Our study is consistent with previous literature in this area [2, 12]. Evans et al. showed similar patterns using aggregate billing data. When billing patterns are broken down by class, in both Evans et al. and our study, first-year residents’ patterns were the closest to those of the attendings [12]. Contrary to the assumption that billing is a skill that develops over time, our study provides evidence that performing billing tasks is likely to be also influenced by yet-to-be-identified intrinsic factors (e.g., willingness to staff visits in real time, asking for help when uncertain) and extrinsic factors (e.g., time constraints, number of patients).

Previous research has focused on financial effects of residents’ billing patterns, claiming that lower billing code use results in tens of thousands of dollars in lost revenues [2, 12], contributing more damage to already fragile bottom lines. The often-unspoken consequence of misclassifying codes is the effect on patient care and safety, which can be influenced by the level of attending physician direct or indirect involvement. If the visit is classified as requiring more complex decision-making (i.e., 99,214–5), the attending must *directly* participate in aspects of patient care such as obtaining the history, examining the patient, and making decisions. In contrast, if the visit is classified as requiring less complex decision-making (i.e., 99,212–3) only *indirect* attending participation is required. When indirectly involved, the attending relies on the resident’s assumed competency, sometimes only providing instructions after the patient has left. Whether to involve the attending physician is a judgment call made by the resident. As our study indicates residents may bill lower E/M codes more often than they should, we also suggest that hundreds or thousands of patient visits may be classified with lower complexity when, in fact, they should have direct attending involvement.

The patterns observed in relation to gender are particularly interesting. In our study’s unique context, male attendings billed higher E/M codes more frequently than female attendings, while female residents billed higher E/M codes more frequently than male residents. These statistically significant findings may invite speculation about variations in male and female risk aversion and norm adherence [20]. It may also, if proven consistent in future studies, come to partially explain variations in male and female provider compensation [21–26]. However, considering the scope and primary aims of the present study, such suggestions should be taken with caution. It is our position that gender is a complex concept, and our identified association should not be interpreted simplistically as causation, such as “a person bills this way because they are male or female.” Nonetheless, it is an invitation for conversation and exploration of the role of gender in actions like coding and billing, which present judgments of complexity and value of someone’s work.

Our study has many practical implications for teaching and practice management. Strategies can be implemented to address variations in coding, for example, before-clinic huddles, where residents and attendings review patients, identifying those requiring direct supervision (i.e., coded 99,214 or higher). As our study demonstrates, administrative data can provide a bird’s eye view of resident and attending billing patterns. These data can be exploited for educational purposes such as unexplained resident or attending deviations from expected billing patterns can be called out and remediated. Finally, closer looks at coding patterns using tools like chart audits or observations (video-mediated or with the attending present) can facilitate access to what took place and allow for better conversations and closer involvement of attending physicians in complex cases. These strategies are being explored in resident training at the institution where the study was conducted. Since coding is a hands-on skill, it is better learned with practice, reflections, and conversations with more skilled attendings or coding experts.

Our study has several strengths. First, unlike previous work that relied only on descriptive, aggregate data, we used visit-level information, allowing for adjustment of multiple provider and patient characteristics and assuring the robustness of our conclusion. Second, we compared the behaviors of attendings and resident groups over a relatively long period (6 years) in a single, relatively stable clinical setting. This advantage allowed many observations to be gathered, while simultaneously limiting the likelihood of wide, unmeasured differences between patient populations. Third, to assess effects of years of training, we used a cohort study design, which is appropriate for assessing changes over time and is less susceptible to unmeasured variations that may have affected previous cross-sectional designs.

Despite its strengths, our study has limitations. First, while our data spans many years, they are limited to one residency. This prohibits generalizability of our findings to residencies with different practice models, including those not applying primary care exception for all patients or not incentivizing attendings’ clinical work and those where residents have limited billing roles. Second, while we considered many important factors, others were omitted due to the study’s nature. For example, we considered attendings as a homogenous group despite recognition that billing patterns may vary with years of experience, other training, and areas of concentration. Third, without a gold standard, our study is a mere comparison between groups and we could not say if one group billed lower than the standard or the other group billed higher. Finally, differences in age and disease prevalence of patients seeing residents versus attendings are significant. Regression models, however, adjust for some of these differences, and stratified analysis by age showed patterns consistent with our main finding.

## Conclusion

Our study confirms that, overall, residents are less likely than attendings to select higher billing codes in similar visits. Care provider gender, years of training, patient age, medical conditions, and number of addressed problems are all implicated in coding and billing variations. While these patterns of billing are known to contribute to lost revenues, further studies are required to explore their effects on resident supervision and patient care.

## Abbreviations

ICD-9: International classification of diseases 9th revision
IRB: International review board.
